# Microbiome engineering: engineered live biotherapeutic products for treating human disease

**DOI:** 10.3389/fbioe.2022.1000873

**Published:** 2022-09-16

**Authors:** Jack W. Rutter, Linda Dekker, Kimberley A. Owen, Chris P. Barnes

**Affiliations:** ^1^ Department of Cell & Developmental Biology, University College London, London, United Kingdom; ^2^ Department of Genetics, Evolution & Environment, University College London, London, United Kingdom

**Keywords:** microbiota, microbiome engineering, human health, clinical trials, engineered bacteria

## Abstract

The human microbiota is implicated in many disease states, including neurological disorders, cancer, and inflammatory diseases. This potentially huge impact on human health has prompted the development of microbiome engineering methods, which attempt to adapt the composition and function of the human host-microbiota system for a therapeutic purpose. One promising method is the use of engineered microorganisms that have been modified to perform a therapeutic function. The majority of these products have only been demonstrated in laboratory models; however, in recent years more concepts have reached the translational stage. This has led to an increase in the number of clinical trials, which are designed to assess the safety and efficacy of these treatments in humans. Within this review, we highlight the progress of some of these microbiome engineering clinical studies, with a focus on engineered live biotherapeutic products.

## 1 Introduction

The human microbiota refers to all microorganisms and microbial communities that colonise the human body. The largest of these communities is found within the digestive tract. However, there are many other communities that play a role in human health, including the skin (Byrd et al., 2018), vaginal (Chen et al., 2021), and oral microbiota (Lamont et al., 2018). The microbiota potentially plays an integral role in human disease, and dysbiosis in these communities has been implicated in numerous conditions. To date, the microbiota has been linked to neurological diseases (*via* the gut-brain axis) (Morais et al., 2021; Liu et al., 2022), inflammatory diseases (such as ulcerative colitis) (Caruso et al., 2020), and cancer (Helmink et al., 2019), amongst others (Pascal et al., 2018; Gurung et al., 2020; Hao et al., 2021). It is this profound effect that the microbiota is thought to impart on the human host that has driven interest in microbiome engineering methods, which aim to modify the human host-microbiota system for a therapeutic purpose; particularly for diseases where current treatment is inadequate or non-existent (Brennan, 2022).

This review focuses on studies that use recombinant or engineered live biotherapeutic products (eLBPs); in particular, highlighting clinical trials that attempt to demonstrate their safety and efficacy in humans. eLBPs are microorganisms that have been genetically modified to perform a specific diagnostic or therapeutic function. Previous reviews have summarised the progress that has been made in developing these methods, but the majority focus on preclinical research, with only a few referring to clinical trials (Riglar and Silver, 2018; Charbonneau et al., 2020; Cubillos-Ruiz et al., 2021; McNerney et al., 2021). In this review we firstly introduce the concept of eLBPs and how they differ from other microbiome engineering techniques, before discussing relevant clinical trials that are using eLBPs.

## 2 Engineered live biotherapeutic products

**FIGURE 1:**
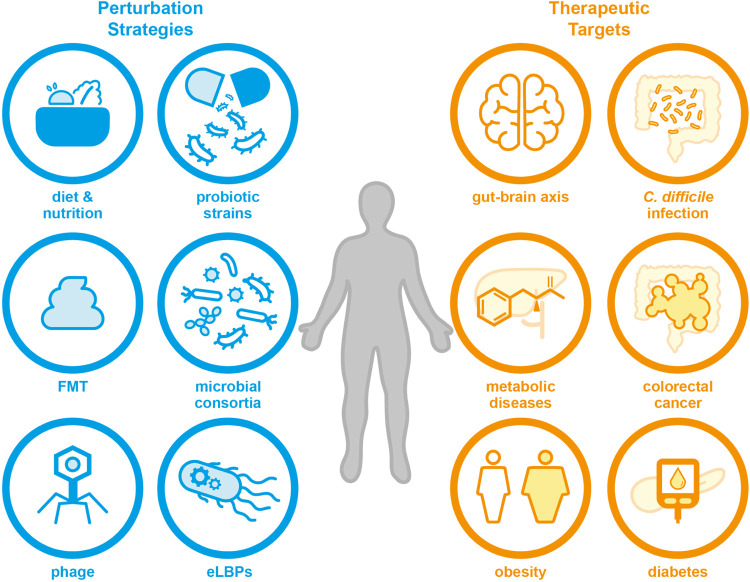
There are many techniques that can be used to engineer the human microbiota towards a therapeutic purpose, including probiotic strains, defined microbial consortia, personalised diet and nutrition, or eLBPs (which are the focus of this review). These methods may be useful for the treatment of a range of human diseases in future, for example metabolic diseases, diabetes and neurological conditions.

Although the human microbiota is an incredibly complex community, there are many strategies that can be used to try and influence its composition and/or function (Lawson et al., 2019; Madhusoodanan, 2020). Some examples of previously reported strategies are given in Figure 1. One common method uses naturally occurring probiotic bacteria, which are thought to confer a health benefit to the host; although often their effects on the host and native microbiota are not fully understood and evidence of their ability to effectively colonise the gut remains sparse (Liu et al., 2018; Zmora et al., 2018; Wieërs et al., 2020). It should be noted that this short residency time can also occur with eLBPs depending on the host chassis chosen, as shown by an engineered *E. coli* Nissle 1917 strain that had a mean residence time of 48 h in human patients (Kurtz et al., 2019). Although this may be desirable in certain cases as it allows for more reproducible and predictable pharmacological properties (Adolfsen et al., 2021), repeated dosing may be required in order to provide prolonged residency of the therapeutic strain (Charbonneau et al., 2020). In addition, clinical evidence implies that the effectiveness of probiotics can vary, whereby some patients may benefit from treatment whilst others do not (Reid et al., 2010). Over 1,000 clinical studies have involved probiotics (Dronkers et al., 2020), exploring conditions such as Parkinson’s disease (NCT04389762) (Lu et al., 2021), COVID-19 infection (NCT04399252) (Tang et al., 2021), and atopic dermatitis (NCT02585986) (Climent et al., 2021). Additionally, it is possible to use multiple species as part of natural or synthetic consortia. Faecal microbiota transplants (FMTs) are a prominent example, where a “healthy” individual’s faecal matter is used to try and replace a patient’s dysbiotic microbiota (Gupta et al., 2016). Currently, there are many clinical trials exploring FMTs and the FDA permits their use under “enforced discretion” for *Clostridioides difficile* infection (CDI) that does not respond to standard therapy (Grigoryan et al., 2020). Other perturbation strategies include synbiotics (Panigrahi et al., 2017), phage (Schooley et al., 2017), diets and personalised nutrition (e.g. NCT01892956) (Zeevi et al., 2015). A non-exhaustive list of clinical trials investigating various microbiome engineering techniques is given in SI table 1.

**FIGURE 2:**
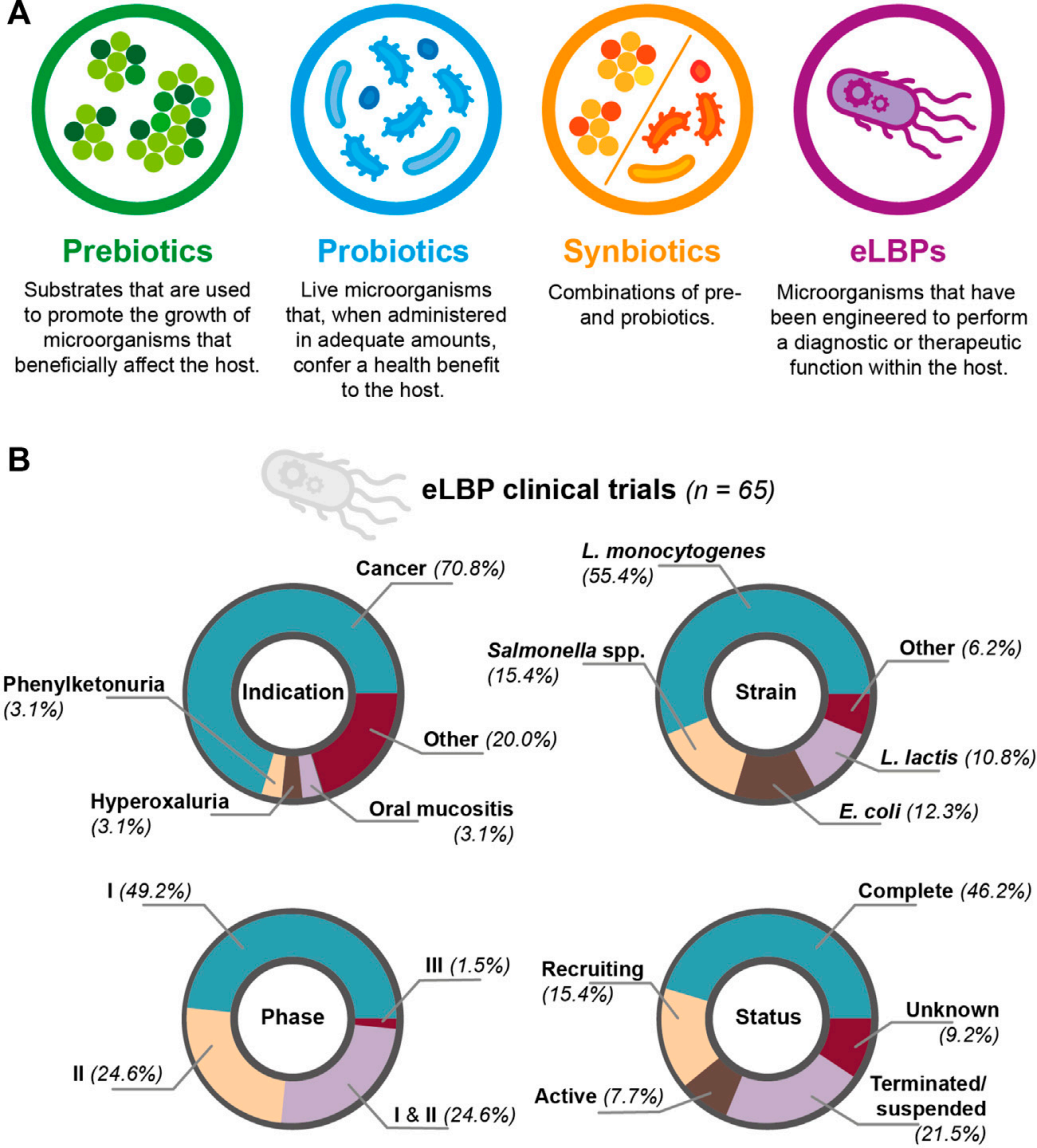
**(A)** Comparison of prebiotics, probiotics, synbiotics and eLBPs. All offer promising routes for modifying the human microbiotia **(B)** Summary of clinical trials involving eLBPs (genetically-modified microorganisms used for a therapeutic purpose), discovered during this literature review.

Engineered methods include eLBPs (Ozdemir et al., 2018; Tan et al., 2020). A simple illustration of this strategy is engineering a bacterial chassis to express a therapeutic molecule. In a ground-breaking study, Steidler et al. (2000) demonstrated this principle by engineering *Lactococcus lactis* to produce interleukin-10 for the treatment of murine colitis (Steidler et al., 2000). In a more recent example, Cubillos-Ruiz et al. (2022) engineered *Lactococcus lactis* to produce a heterodimeric *β*-lactamase, showing that the strain was able to reduce ampicillin-induced dysbiosis in a mouse model (Cubillos-Ruiz et al., 2022). Using eLBPs offers several advantages over the use of pre-, pro- or synbiotics (as defined in Figure 2A), as genetic engineering can confer functions that are not expressed by the native microbiota (Cubillos-Ruiz et al., 2021). Other advantages include the ability to choose a defined strain chassis, reducing the possibility of introducing pathogenic species and the secretion of non-native molecules (Charbonneau et al., 2020; Yadav and Chauhan, 2022). Furthermore, additional genetic elements (e.g. auxotrophies and inducible promoters) allow for greater control and flexibility of eLBPs in the host and environment (Brennan, 2022). For example, Harimoto et al. (2022) developed a programmable surface capsular polysaccharide (CAP) system in *Escherichia coli* Nissle 1917; through inducible expression the CAP system controlled bacterial encapsulation and subsequently, showed a ten-fold increase in the maximum tolerated dose and anti-tumour efficacy of the strain in a mouse model of cancer (Harimoto et al., 2022). However, with prolonged use of eLBPs there are concerns regarding loss of function, mutation, and biocontainmnent of both the whole organism and genetic material (Ozdemir et al., 2018; McNerney et al., 2021). In addition, outside of *E. coli* and *Lactobacillus*, there are relatively few tools available for engineering other relevant species (Charbonneau et al., 2020; Chen et al., 2021). To date, the vast majority of these methods have only been tested in *vitro* or animal models. Examples of these studies are provided in SI table 2. In recent years, an increasing number of eLBPs have entered clinical trials resulting in more data on the safety and efficacy of eLBPs in humans. Through the clinicaltrials.gov database and literature review, we identified 65 clinical trials involving eLBPs, although others may exist. Of these studies, 46.2% were reported as complete, 21.5% as terminated and the remainder as recruiting, active or unknown (Figure 2B). Within this review, we primarily focus on two major areas of research: 1) cancer therapeutics, and 2) metabolic diseases.

## 3 Cancer therapeutics

Treating cancer with bacteria is not a new concept. One of the earliest reported cases of immunotherapy dates back to the 19th century when mixtures of heat-killed bacteria, known as “Coley’s toxin”, were used to treat inoperable tumours (Coley, 1891; Bickels et al., 2002). Although not without criticism, as little was known about the underlying principles by which it worked, many patients were declared disease free after treatment (McCarthy, 2006). In addition, many species of bacteria have an inherent ability to preferentially colonise within the tumour microenvironment (Duong et al., 2019). It has also been shown that different tumour types have distinct microbiota compositions (Nejman et al., 2020) and that the gut microbiota is able to modulate the effects of chemotherapeutic drugs (Heshiki et al., 2020). These factors have led to interest in harnessing bacteria as vaccine products or as vehicles for the production of localised therapeutics directly within the tumour (Chowdhury et al., 2019). Therefore, it is of little surprise that cancer therapy is one of the most common targets for eLBP clinical trials (Figure 2B).

Currently, most cancer-focussed eLBPs in clinical trials can be grouped into two categories based on the chosen chassis: trials using *Listeria monocytogenes* (36 trials) or a *Salmonella* subspecies (10 trials). Attenuated strains of *L. monocytogenes* have been produced that display reduced virulence. For example, the double-knockout Δ*actA*/Δ*inlB* strain, where the genes for tropism and cell-to-cell transmission have been deleted; or the Δ*actA*/Δ*plcB* strain (Johnson et al., 2011; Flickinger et al., 2018). These attenuated strains are commonly engineered to act as vaccines for specific forms of cancer, via secretion of a target antigen (Morrow et al., 2019). A prominent example is ADXS11-001 (also known as AXAL, or ADXS-HPV), an eLBP developed by Advaxis for the treatment of HPV-associated cancers (Galicia-Carmona et al., 2021). This *prfA*-defective strain secretes a truncated fragment of listeriolysin O (LLO), an immunological pore-forming protein, fused to HPV-16 E7 (Miles et al., 2017). The ADXS11-001 strain has been involved in several clinical trials for the treatment of cervical cancer (e.g. NCT02164461, and NCT01266460). In addition, ADXS11-001 has been evaluated as a treatment for anal/rectal (NCT02399813), head and neck cancers (NCT02002182). Currently, the AIM2CERV double-blind, placebo-controlled randomised study investigating ADXS11-001 for high risk cervical cancer, is the only phase III clinical trial for a *L. monocytogenes* based eLBP (NCT02853604) (Flickinger et al., 2018). However, not all ADXS11-001 trials have been successful, as a phase I trial (NCT01598792) for oropharyngeal cancer was terminated early after a patient suffered from dose-limited toxicity (Sacco et al., 2016).

Advaxis also developed the ADXS31-142 and ADXS31-164 eLBPs. ADXS31-142, intended for the treatment of prostate cancer, was engineered to secrete a fusion of LLO and prostate specific antigen (a serine protease that displays elevated levels during prostate cancer progression) (Hannan et al., 2012). Whereas ADXS31-164 was engineered to secrete an LLO-HER2/neu chimeric protein (Shahabi et al., 2011). As with ADXS11-001, both of these products have now gone through phase I/II clinical trials (NCT02325557 and NCT02386501, respectively).

*Salmonella* subspecies are also commonly used, as they can penetrate and preferentially grow within tumour tissues (Pangilinan and Lee, 2019). The VXM01 strain, developed by Vaximm using a *Salmonella typhi* chassis, was engineered to produce vascular endothelial growth factor-2 (Wick et al., 2018). This strain was initially trialed with 45 patients suffering from advanced/stage IV pancreatic cancer (NCT01486329) (Niethammer et al., 2012). A further phase I clinical trial (NCT02718443) tested an oral administration of VXM01 on 14 patients with glioblastoma. VXM01 was found to produce a positive increase in the CD8/Treg ratio of post-vaccine tumour tissues (Wick et al., 2018). Following these promising results, a phase I/II clinical trial (NCT03750071) is now recruiting 30 patients with recurrent glioblastoma. This study will explore the efficacy of VXM01 in combination with Avelumab (a checkpoint inhibitor), following standard treatment. Further trials are exploring the use of engineered *Salmonella* for the treatment of liver cancer (NCT01099631), myeloma (NCT03762291), and neuroblastoma (NCT04049864).

Alongside the *L. monocytogenes* and *Salmonella* based studies, *E. coli* Nissle 1917 has been used as a chassis for cancer-targeting eLBPs. Synlogic’s SYNB1891 strain was engineered to target the stimulator of interferon genes (STING) agonists pathway to trigger anti-tumour immunity, via expression of cyclic di-AMP (CDA) (Leventhal et al., 2020). Alongside CDA production, a biocontainment system was added to the SYNB1891 strain. Leventhal et al. introduced Δ*dapA* (4-hydroxy-tetrahydropicolinate synthase) and Δ*thyA* (thymidylate synthesase) knockouts, showing that this dual-auxotroph was able to prevent *in vivo* proliferation of SYNB1891 in various tumour types (Leventhal et al., 2020). SYNB1891 was able to stimulate anti-tumour immunity in tumour-bearing mice and triggered the STING pathway in human antigen-presenting cells *in vitro*. SYNB1891 has subsequently entered a phase I clinical trial (NCT04167137). This trial is recruiting patients with advanced/metastatic solid tumours and lymphoma, with the aim to test SYNB1891 alone or in combination with Atezolizumab (an immunotherapy drug). These studies highlight the vast potential eLBPs hold for the treatment of a variety of cancer types.

## 4 Metabolic diseases

Synthetic biology engineering techniques allow for the creation of eLBPs that can produce non-native molecules, such as human proteins (Charbonneau et al., 2020). This allows for eLBPs that can correct conditions caused by errors of human metabolism. There are several promising eLBPs for treating metabolic diseases in clinical development. For example, the SYNB1618 *E. coli* Nissle 1917 strain developed by Synlogic, for the treatment of phenylketonuria (PKU). PKU is a rare disease caused by genetic mutations of the phenylalanine hydroxylase enzyme; which results in elevated levels of phenylalanine in the blood. If left untreated, PKU can cause severe neurological complications. Traditionally, PKU is managed through a phenylalanine-restricted diet, which can have a negative impact on a patient’s quality of life (Rocha and MacDonald, 2016). The SYNB1618 strain was engineered to consume phenylalanine within the digestive tract, via action of the phenylalanine ammonia lyase (PAL) and l-amino acid deaminase (LAAD) enzymes. Following positive results in mouse and non-human primate models of PKU (Isabella et al., 2018), SYNB1618 was evaluated in a Phase I/IIa clinical study (NCT03516487). This trial recruited both healthy volunteers and PKU patients; showing that SYNB1618 was safe and well-tolerated up to a maximum dose of 2 × 10^11^ colony-forming units (Puurunen et al., 2021). As discussed by the authors, the successful completion of this clinical trial provided proof of the potential to use eLBPs in the treatment of rare metabolic conditions (Puurunen et al., 2021). In addition, data from this study has been used to develop *in vitro* gut-on-chip (Nelson et al., 2021) and predictive pharmacology models (Charbonneau et al., 2021); showing that results from clinical trials can feed back into the preceding laboratory design process, in order to help address some of the limitations present in animal models (Brennan, 2022). Following the successful trial of SYNB1618, Synlogic developed the SYNB1934 strain (Adolfsen et al., 2021). SYNB1934 was created through optimisation of PAL enzyme activity, displaying a two-fold increase in *vivo* PAL activity relative to SYNB1618. A phase I clinical trial (NCT04984525) successfully showed that SYNB1934 was also safe and well-tolerated in healthy adult volunteers. Subsequently, a new clinical trial (NCT04534842) is recruiting patients, in order to perform a head-to-head comparison of the SYNB1618 and SYNB1934 eLBPs.

Another *E. coli* Nissle 1917 based eLBP, referred to as SYNB8802, has been designed to combat enteric hyperoxaluria (EH). EH is caused by excessive absorption of dietary oxalate, which can cause kidney failure. This can be due to genetic defects, or via gastrointestinal conditions that increase oxalate uptake (Witting et al., 2021). Currently, there are no approved pharmaceutical treatments for this condition (Lubkowicz et al., 2022). SYNB8802 was modified to express genes from the oxalate degradation pathway of *Oxalobacter formigenes*, alongside an oxalyl-CoA synthetase gene. These modifications conferred the ability for SYNB8802 to degrade oxalate *in vitro* (Lubkowicz et al., 2022). As with SYN1618, SYNB8802 was tested in mouse and non-human primate models; demonstrating that SYNB8802 could reduce the excreted levels of urinary oxalate (Lubkowicz et al., 2022). A phase I clinical trial (NCT04629170) is now recruiting healthy adults and EH patients, to evaluate the safety and efficacy of SYNB8802 in humans. Novome Biotechnologies have also developed an engineered *Bacteroides* strain for the treatment of EH, named NOV-001; which is currently recruiting for a phase I/II clinical trial (NCT04909723).

The Synlogic SYNB1020 strain was developed as a treatment option for hyperammonia; a condition caused by liver damage (i.e. cirrhosis), or defects in ammonia-detoxifying enzymes. SYNB1020, another *E. coli* Nissle 1917 strain, was engineered to convert NH_3_ to arginine in anaerobic conditions; with the strain promoting improved survival in a mouse model of hyperammonia (Kurtz et al., 2019). The strain completed phase I clinical trials (NCT03179878). However, SYNB1020 failed a subsequent phase Ib/IIa clinical trial (NCT03447730), which was terminated due to a reported lack of efficacy.

## 5 Other targets

Inflammatory bowel disease (IBD) is a chronic, inflammatory disease of the digestive tract, with two major subtypes: Crohn’s disease and ulcerative colitis (UC). The full mechanisms behind IBD are not yet understood, however there is a general consensus that the microbiota plays an integral role in the development and progression of IBD (Lee and Chang, 2021). Traditionally, IBD therapies attempt to target cytokines of the inflammatory cascade or systemic immunomodulation. However, these treatments are not always effective and may cause adverse side effects (Eindor-Abarbanel et al., 2021). As such, there are several clinical trials exploring the use of probiotics for the treatment of IBD (e.g. NCT04969679, NCT04842149). Additionally, ActoGenix have developed two eLBPs to treat these conditions that have entered clinical trials. The first, AG011, was an engineered *L. lactis* strain that secreted hIL-10 and was shown to be safe and well-tolerated in an UC patient cohort (NCT00729872). AG014, based on the same *L. lactis* platform as AG011, was engineered to produce an anti-TNF*α* antibody fragment of certolizumab (Vandenbroucke et al., 2010). AG014 was evaluated as part of a phase I clinical trial, but has not been developed further (Crowe et al., 2018).

Another eLBP, AG013, was created to treat oral mucositis (OM) in patients with head and neck cancer undergoing chemoradiation therapy. This *L. lactis* strain was engineered to secrete human Trefoil Factor 1. Initially, AG013 was shown to be effective in a hamster based model of radiation-induced OM (Caluwaerts et al., 2010). Following a successful phase I trial (NCT00938080), which demonstrated the safety of AG013 in patients receiving induction chemotherapy for the treatment of head and neck cancer, AG013 entered phase II clinical trials (NCT03234465). However, in a statement issued by Oragenics, Inc. this study was terminated as there was no statistically significant difference in the duration of OM between AG013 and the placebo.

## 6 Outlook

Currently, many eLBPs in trials are based on the constitutive expression of specific molecules. However, modern engineering tools allow for the development of more complex systems. For example biosensors that sense and respond to environmental cues in a dynamic manner (Riglar et al., 2017; Hicks et al., 2020; Rutter et al., 2021), engineered strains that display targeted or prolonged colonisation in a specific environment (Fedorec et al., 2021; Chien et al., 2022), and living materials that are embedded with engineered microbes (Rodrigo-Navarro et al., 2021; Omer et al., 2022). Recent examples include biosensors for acetoacetate, oxygen lactacte, pH and inflammation (Riglar et al., 2017; Rutter et al., 2021; Chien et al., 2022); ingestible micro-bio-electronic devices that use heme-sensitive bacteria to report on gastrointestinal health (Mimee et al., 2018); and engineered native strains that were used for prolonged transgene delivery in a mouse model (Russell et al., 2022). Furthermore, several methods for mitigating the issue of biocontainment for eLBPs have been developed. One method that has already been used in clinical trials are auxotrophies that attempt to limit the growth of engineered strains outside of the desired environment, for example the Synlogic Δ*dapA* auxotroph for the essential cell wall component diaminopimelate (Adolfsen et al., 2021). Other biocontainment strategies include ‘deadman’ and ‘passcode’ kill switches, which can be re-programmed for various environmental cues. These switches can be used to block the transcription of essential genes in the absence of a specific input or to trigger self-killing of engineered strains via the production of a toxin, preventing undesired cell growth (Chan et al., 2016). We expect clinical trials evaluating these more complex systems to arise; although it will be essential to ensure these systems behave in a predictable and robust manner. Furthermore, it should be noted that an integral feature of eLBPs is that a single strain can be engineered to target more than one condition simultaneously (Brennan, 2022). As stated by Puurunen et al. (2021) this is particularly promising for the treatment of metabolic diseases and differentiates eLBPs from traditional methods, such as enzyme replacement therapy (Puurunen et al., 2021). Therefore, it is possible that more advanced eLBPs, which are able to produce multiple therapeutic effectors from a single strain, will enter clinical trials.

As evidenced by the number of complete, and ongoing, clinical trials, the development of eLBPs is an exciting application of microbiome engineering research. Despite this, it is important to highlight the issues that can occur when eLBPs enter clinical trials. As shown by the number of terminated and unsuccessful clinical trials, a major issue is that positive *in vitro* and animal model data do not always translate into safe and efficacious treatment in humans. Of the 14 terminated eLBP clinical trials identified during this literature review 50% were terminated due to a reported lack of efficacy or clinical activity in humans. Although undoubtedly more needs to be done to alleviate these problems, considerable research is going into the development of novel model systems that can be used to characterise eLBPs more comprehensively before they reach clinical testing. These include novel animal models (Hwang et al., 2017; Rutter et al., 2019), innovative organ-on-chip systems that try to mimic human tissues (Bein et al., 2018; Nelson et al., 2021) and mechanistic models that attempt to predict the *in vivo* activity of eLBPs (Charbonneau et al., 2021). It is hoped that these new technologies will benefit the patients that need these therapies most by helping to translate some of the many developed eLBP strains into clinical treatments.

Finally, as discussed in detail by Charbonneau et al. (2020), it is important to consider the wider regulatory, environmental and societal impacts these new treatment modalities may have if they are to be approved for human use (Charbonneau et al., 2020). For example, manufacturing facilities will be needed that are able to produce standardised, high-quality eLBPs at large scales, while complying with current good manufacturing practices (cGMP) (Brennan, 2022). In addition, physicians, patients and the general public will need to be educated on the benefits and associated risks of these products if they are to become widely accepted (Charbonneau et al., 2020). Although it is evident that challenges remain, eLBPs hold vast potential to address unmet needs in the treatment of human disease. It will be interesting to watch how society reacts to these technologies as they continue to approach the clinic over the coming years.
